# Effectiveness and safety of bivalirudin anticoagulation therapy in adult patients receiving extracorporeal membrane oxygenation: A systematic review and meta-analysis

**DOI:** 10.1097/MD.0000000000042696

**Published:** 2025-08-15

**Authors:** Yunnan Hu, Linchang Yu, Wendian Yang

**Affiliations:** a Emergency Department, Shenzhen Longgang Second People’s Hospital, Shenzhen, Guangdong Province, China; b Intensive Care Medicine Department, Shenzhen University General Hospital, Shenzhen, Guangdong Province, China.

**Keywords:** bivalirudin, ECMO, heparin, meta-analysis

## Abstract

**Background:**

Compared to heparin, there is limited evidence of the benefits of bivalirudin in the patients with extracorporeal membrane oxygenation (ECMO).

**Methods:**

We searched for studies comparing bivalirudin anticoagulation with heparin anticoagulation in ECMO patients in the PubMed, Embase, and Cochrane Library databases from inception to March 28, 2024. ECMO patients were divided into the bivalirudin experimental group and the heparin control group. Collected data were mortality rate, duration of ECMO, successful decannulation rate, incidence of thrombotic events, incidence of bleeding events, activated partial thromboplastin time values, platelet transfusion requirements, and other indicators for ECMO patients in the bivalirudin and heparin groups. StataMP17 and RevMan 5.4 software for data analysis and visualization were used.

**Results:**

A total of 11 papers that met the inclusion criteria were included. Compared to the traditional heparin anticoagulation group, the bivalirudin anticoagulation group had a lower mortality rate (odds ratios [OR] = 0.74, 95% confidence intervals [CI; 0.56, 0.99], *Z* = 2.01, *P* = .04) and incidence of thrombotic events (OR = 0.52, 95% CI [0.38, 0.71], *Z* = 4.11, *P* < .0001); meanwhile, the ECMO decannulation success rate (OR = 1.87, 95% CI [1.2, 2.92], *Z* = 2.75, *P* = .006) was higher, with statistically significant differences between the 2 groups. In ECMO patients, there were no significant differences between bivalirudin and heparin in terms of ECMO duration, incidence of bleeding events, platelet transfusion requirements, and activated partial thromboplastin time, with no statistical significance between the 2. Subgroup analysis suggested that the mortality rate in the veno-venous group was lower than that in the control group, but the difference was not statistically significant (OR = 0.52, 95% CI [0.27, 0.99], *Z* = 1.99, *P* = .05). The mortality rate in the non-veno-venous group was lower than that in the control group, with no statistically significant difference (OR = 0.82, 95% CI [0.61, 1.11], *Z* = 1.26, *P* = .21).

**Conclusion:**

Bivalirudin anticoagulation significantly reduced the mortality rate and incidence of thrombotic events in ECMO patients, while significantly increasing the success rate of ECMO decannulation. However, due to clinical heterogeneity and institutional variability in monitoring and transfusion practices, these findings should be interpreted with caution. Further stratified research is needed to confirm these outcomes across different ECMO subgroups.

## 1. Introduction

Extracorporeal membrane oxygenation (ECMO) is an advanced life support technology in intensive care medicine that plays a crucial role in treating critically ill patients. ECMO is typically categorized into 2 primary modes: veno-venous (VV) ECMO, which is used primarily for respiratory failure, and veno-arterial (VA) ECMO, which provides both respiratory and cardiac support. The choice of anticoagulant in these settings may depend on various factors, including the type of ECMO support, the patient’s underlying conditions, and the specific risks associated with each mode. The principle of ECMO involves withdrawing the patient’s blood from the body via a pumping device, transporting it through tubing to an oxygenator where oxygenation and carbon dioxide removal occur, and finally returning the blood to the patient’s body.^[1]^ By this principle, ECMO can provide respiratory and cardiac support in certain extreme cases, maintaining stability in respiratory and circulatory functions to ultimately save lives. ECMO has a wide range of clinical applications, including but not limited to acute respiratory distress syndrome, cardiac arrest, post-acute myocardial infarction shock, and organ transplantation. Research indicates that ECMO, when added to conventional cardiopulmonary resuscitation, significantly improves the success rate of resuscitation.^[2]^ In these critical situations, ECMO provides sufficient oxygenation and circulatory support to help patients through the danger zone, increasing survival rates.

During ECMO, the patient’s blood comes into contact with various artificial materials and surfaces of devices such as oxygenators, making it prone to triggering the coagulation system and causing thrombus formation. Therefore, anticoagulants are used during ECMO therapy to prevent thrombosis.^[3]^ Heparin, due to its low cost and effective anticoagulant function, is commonly used in ECMO patients. The principle of heparin involves binding to antithrombin III, thereby inhibiting the activity of thrombin, preventing blood clotting, and ensuring smooth blood circulation.^[4]^ However, heparin has several disadvantages in anticoagulation. Firstly, heparin often causes a decrease in platelet count, possibly due to the formation of immune complexes between heparin and platelet factors, triggering an immune reaction.^[5]^ Secondly, heparin’s anticoagulant status is unstable, and some patients may experience unstable coagulation due to heparin allergy or reduced tolerance.^[6]^ Lastly, heparin only inhibits thrombin III, which may not be effective for patients with deficiencies in thrombin III.^[7]^

Due to these shortcomings of heparin, there is a need to study novel anticoagulants. However, there are specific situations in which heparin may still be necessary, such as in cases of resistance to direct direct thrombin inhibitors (DTIs) or when additional anticoagulation laboratory monitoring is required to ensure therapeutic anticoagulation levels. These factors must be carefully considered when choosing an appropriate anticoagulant for ECMO patients.

Bivalirudin, as a DTI, can replace heparin in anticoagulation therapy for ECMO patients. Bivalirudin has advantages such as a short half-life, independence from antithrombin III, and no significant decrease in platelets.^[8]^ Despite these advantages, there is still some controversy over whether bivalirudin is preferred to heparin. Our research aims to compare the effectiveness and safety of bivalirudin versus heparin during ECMO support to determine which anticoagulant is preferred.

Our research aims to compare the effectiveness and safety of bivalirudin versus heparin during ECMO support to determine which anticoagulant is preferred. In doing so, we also aim to highlight methodological limitations, such as population heterogeneity and differences in ECMO modalities, which can influence interpretation of pooled outcomes.

## 2. Materials and methods

### 2.1. Literature search strategy

All articles were searched in the PubMed, Embase, and Cochrane Library databases. The search strategy for the keywords used in PubMed is provided in the Supplementary Materials (Supplemental Digital Content, https://links.lww.com/MD/P743). The search period was from inception to March 28, 2024. This study is registered on the PROSPERO website with the registration number CRD42024530920.

### 2.2. Inclusion and exclusion criteria

#### 2.2.1. Inclusion criteria

Study design: Retrospective study; Study population: Patients aged ≥18 years undergoing ECMO therapy; Intervention: Patients in the experimental group received bivalirudin for anticoagulation, while patients in the control group received standard heparin for anticoagulation and; Outcome measures: Mortality rate, duration of ECMO support, success rate of ECMO decannulation, incidence of thrombotic events, incidence of bleeding events, activated partial thromboplastin time (APTT), platelet transfusion volume.

#### 2.2.2. Exclusion criteria

Studies that do not meet the research objectives; duplicate studies; comments, brief reports, conference abstracts, and case reports; literature in languages other than Chinese or English; animal experiments; and pediatric research.

### 2.3. Literature screening

Throughout the literature screening process, 2 professional researchers were initially selected to conduct preliminary screening of the articles based on their titles and abstracts. Articles that were not relevant to the study were excluded during the initial screening. Subsequently, full-text reading of articles meeting the inclusion criteria was performed for further selection. In the case of discrepancies between the 2 researchers during the screening process, a third reviewer evaluated these articles and made the final decision on their inclusion in the study. Our study adhered to the Preferred Reporting Items for Systematic Reviews and Meta-Analyses (PRISMA) statement. Since all the articles included in this meta-analysis were previously published retrospective studies, ethical review board approval was not required.

### 2.4. Quality assessment of the literature

For all included studies, we assessed the risk of bias using the Newcastle-Ottawa scale, which involves 7 different items. Scores range from 5 to 9 points.

### 2.5. Statistical methods used

We summarized the obtained results using the Mantel–Haenszel random-effects model. When the data were in count format, we analyzed them using odds ratios and 95% confidence intervals (CI). For continuous data, we typically used the mean difference and 95% CI for the analysis. We weighted the studies using inverse variance. We assessed the heterogeneity of the included studies using Cochran’s *Q* statistic and the *I*^2^ test (*P* < .1 or *I*^2^ > 50% indicating significant heterogeneity). Additionally, we conducted sensitivity analyses by sequentially excluding each study to evaluate its impact on the overall results. Subgroup analyses were performed based on the mode of ECMO (VV vs non-VV). Publication bias was assessed using Egger’s test and funnel plots. A significance level of *P* < .05 was considered statistically significant. Data analysis and plotting were performed using RevMan 5.4 and StataMP17 software.

## 3. Results

### 3.1. Article retrieval results and quality analysis

**Figure 1.**
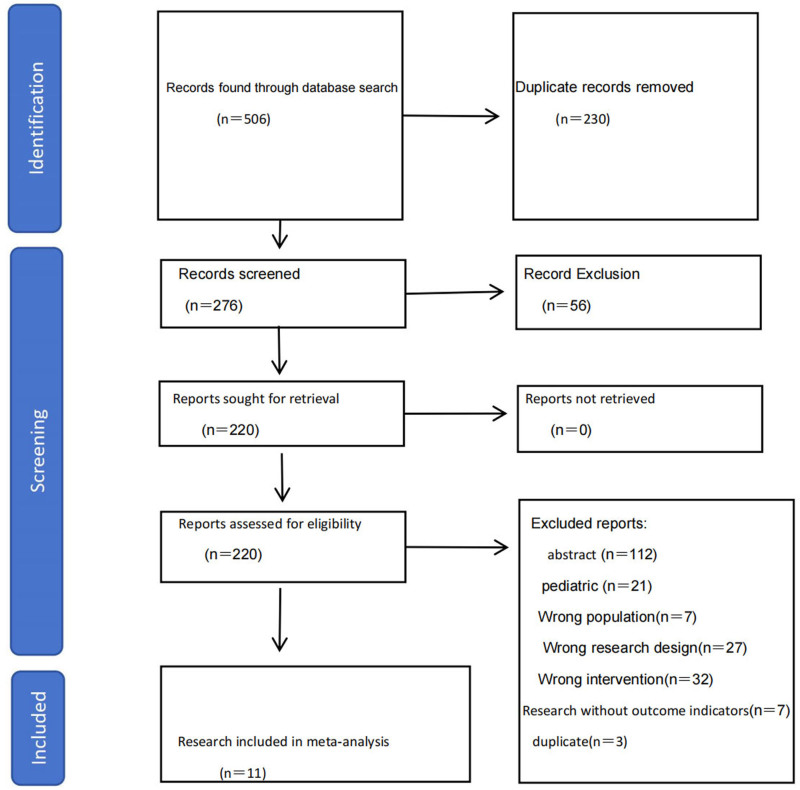
Study flow diagram. Flowchart depicting the selection process for studies included in the meta-analysis, following PRISMA guidelines. A total of 506 articles were identified through database searches, with 11 studies ultimately included after screening titles, abstracts, and full texts based on predefined inclusion and exclusion criteria. PRISMA = Preferred Reporting Items for Systematic Reviews and Meta-Analyses.

We used a combination of subject terms and free terms for our search strategy. Initially, we retrieved 506 articles, of which 230 duplicates were removed using the NoteExpress software, leaving 276 articles. Subsequently, after reviewing the titles and abstracts, we excluded 56 irrelevant studies, resulting in 220 articles. After full-text reading, we further excluded 209 articles. Among these, 112 were unrelated to the study topic, 21 involved pediatric populations, 7 had population errors, 27 had incompatible study designs, 32 had intervention errors, 7 lacked outcome measures, and 3 were duplicates. Finally, we collected 11 articles,^[9–19]^ as shown in Figure 1. These articles included a total of 1360 patients. Among the included articles, 3 had scores of 6 points, while the remaining 8 articles had scores of 7 points on the assessment scale. The quality assessment results of the included studies are presented in Table 1. Baseline characteristics of patients included in the bivalirudin group and control group, as well as the outcome measures included in each study, are presented in Table 2.

**Table 1:** Characteristics of the included studies.

Study	n		Age (yr)		Male (%)	ECMO mode (VV/VA)	Dose		Outcome
	Bivalirudin	Heparin	Bivalirudin	Heparin			Bivalirudin	Heparin	
Dayne 2023^[9]^	12	10	43.7 ± 8.148	49.6 ± 11.1	72.7	22/0	0.15 mg/kg/h	12.5 U/kg/h	①②④⑥⑦
Marissa 2023^[10]^	54	89	53 ± 14.815	53 ± 14.815	70.63	0/143	NR	NR	①②③④⑤⑥⑦
Tong 2023^[11]^	14	20	52.82 ± 8.13	50.51 ± 15.52	64.7	34/0	0.025 mg/kg/h	50–100 IU/kg/h	①②③④⑤⑥⑦
Kartika 2023^[12]^	34	61	44 ± 12.1	45 ± 14.2	65.3	95/0	NR	0.20–0.40 U/mL	①②⑤⑥⑦
Berei 2018^[13]^	44	28	55.2 ± 15.2	55.9 ± 13.1	65.2	6/66	0.04 mg/kg/h	8–12 IU/kg/h	①②④⑤⑥⑦
Pieri 2013^[14]^	10	10	55.9 ± 13.1	54 ± 12.7	80	10/10	0.025 mg/kg/h	3 IU/kg/h	①②⑥⑦
Seelhammer 2021^[15]^	110	223	NR	NR	65.2	64/269	0.02–0.15 mg/kg/h	15–20 IU/kg/h	①②④⑤⑦
Rivose 2021^[16]^	133	162	49	49	59.7	295/0	0.3 mg/kg bolus	3000–5000 IU bolus	①②③⑤⑥⑦
Sheridan 2021^[17]^	100	50	54.0 ± 15.0	53.0 ± 14.0	70.7	62/88	0.05–0.1 mg/kg/h	12–18 IU/kg/h	①⑥⑦
Giuliano 2021^[18]^	13	131	49.6 ± 19.1	55.9 ± 15.4	56.7	28/116	NR	NR	①
Kaseer 2020^[19]^	19	33	56	53	71.2	24/28	0.1 mg/kg/h	10 IU/kg/h	①②⑥⑦

Outcomes: ① mortality rate, ② ECMO duration, ③ ECMO successfully weaned, ④ APTT, ⑤ platelet, ⑥ bleeding incidence rate, ⑦ embolism incidence rate.

APTT = activated partial thromboplastin time, ECMO = extracorporeal membrane oxygenation, VV = veno-venous, VA = veno-arterial.

**Table 2:** Quality assessment of included studies.

Study	Selection				Comparability	Outcome			
	Exposed cohort	Nonexposed cohort	Ascertainment of exposure	Outcome of interest		Assessment of outcome	Length of follow-up	Adequacy follow-up	Total score
Dayne 2023^[9]^	1	1	1	1	0	1	1	1	7
Marissa 2023^[10]^	1	1	1	1	0	1	1	1	7
Tong 2023^[11]^	1	1	1	1	0	1	0	1	6
Kartika 2023^[12]^	1	1	1	1	0	1	1	1	7
Berei 2018^[13]^	1	1	1	1	0	1	0	1	6
Pieri 2013^[14]^	1	1	1	1	0	1	1	1	7
Seelhammer 2021^[15]^	1	1	1	1	0	1	1	1	7
Rivose 2021^[16]^	1	1	1	1	0	1	1	0	6
Sheridan 2021^[17]^	1	1	1	1	0	1	1	1	7
Giuliano 2021^[18]^	1	1	1	1	0	1	1	1	7
Kaseer 2020^[19]^	1	1	1	1	0	1	1	1	7

### 3.2. Results of the meta-analysis

#### 3.2.1. Inhospital mortality rate

**Figure 2.**
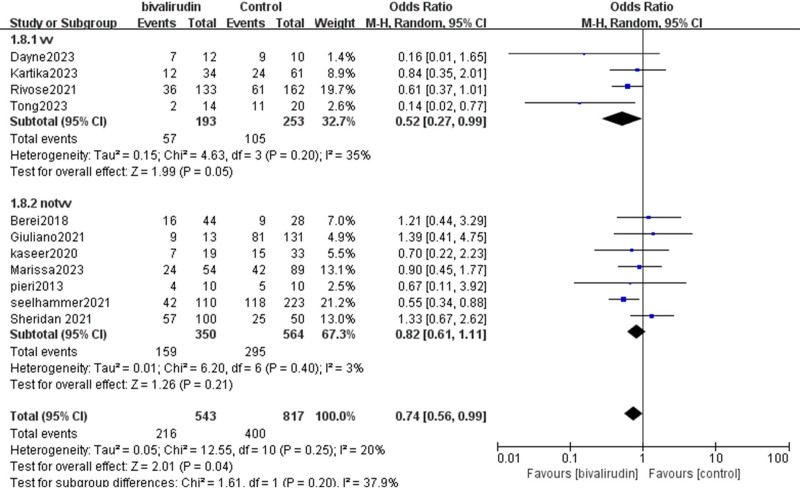
Forest plot of in-hospital mortality comparing bivalirudin and heparin in ECMO patients, with subgroup analysis by ECMO modality. The plot displays overall and subgroup estimates for mortality outcomes. Subgroups include veno-venous (VV) ECMO and non-VV-ECMO (primarily veno-arterial, VA). Odds ratios (ORs) and 95% confidence intervals (CIs) are shown for each study and pooled effects. Labels indicate ECMO type for clarity. CI = confidence intervals, ECMO = extracorporeal membrane oxygenation, ORs = odds ratios, VA = veno-arterial, VV = veno-venous, VV-ECMO = veno-venous extracorporeal membrane oxygenation.

Our study included 11 studies^[9–19]^ that analyzed the impact of bivalirudin versus heparin anticoagulation on in-hospital mortality rates in ECMO patients. The final results showed no significant heterogeneity among the studies (*I*^2^ = 20%, *P* = .25). Our findings indicated that compared to the heparin group, the bivalirudin group significantly reduced the in-hospital mortality rate of ECMO patients, with statistical significance (odds ratios [OR] = 0.74, 95% CI [0.56, 0.99], *Z* = 2.01, *P* = .04), as shown in Figure 2. We performed subgroup analyses of 11 studies based on the cannulation mode of ECMO. Given the different pathophysiological mechanisms underlying VV and VA ECMO, it is essential to consider these differences when evaluating anticoagulation strategies. Veno-venous extracorporeal membrane oxygenation (VV-ECMO) is primarily used for respiratory support, while VA-ECMO is used for both respiratory and cardiac support. Subgroup analysis indicated a trend toward lower mortality in the VV-ECMO group treated with bivalirudin compared to heparin, although the statistical significance was borderline (OR = 0.52, 95% CI [0.27, 0.99], *Z* = 1.99, *P* = .05). For the VA-ECMO (non-VV) subgroup, the mortality reduction was not statistically significant (OR = 0.82, 95% CI [0.61, 1.11], *Z* = 1.26, *P* = .21). It is important to note that the distinct pathophysiological differences and clinical characteristics between VV and VA-ECMO patients introduce significant clinical heterogeneity, which may confound interpretation of pooled outcomes. Therefore, these subgroup findings should be interpreted with caution and should not be generalized across all ECMO modalities..

**Figure 3.**
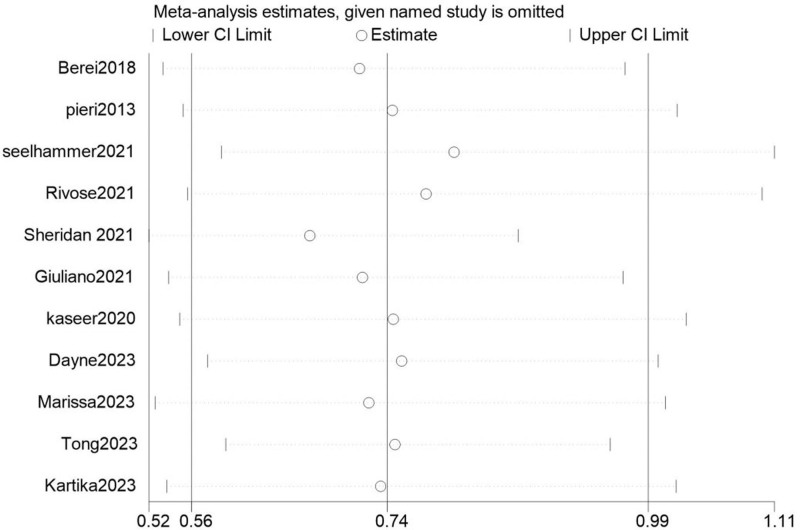
Sensitivity analysis for in-hospital mortality. Sensitivity analysis plot assessing the robustness of the pooled mortality outcome by sequentially omitting each included study. The *x*-axis represents the pooled odds ratios (OR) recalculated after excluding 1 study at a time. Consistent clustering around the central estimate suggests stability of the overall result. CI = confidence intervals, OR = odds ratios.

**Figure 4.**
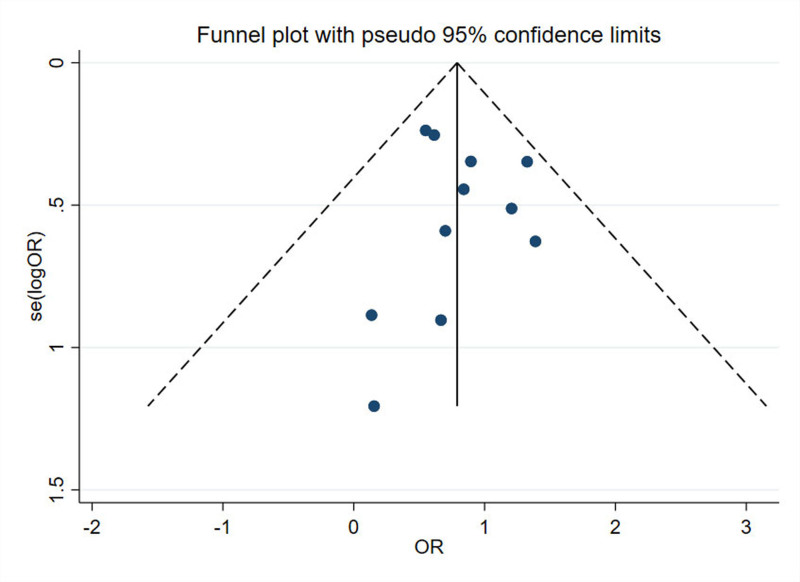
Funnel plot for assessment of publication bias in studies reporting in-hospital mortality. Each point represents an individual study included in the meta-analysis. The symmetrical distribution of studies around the pooled effect estimate suggests a low risk of publication bias. Egger’s test was performed to statistically evaluate asymmetry. ORs = odds ratios.

Subsequently, we conducted a sensitivity analysis by sequentially excluding each of the 11 studies. The sensitivity analysis plot showed that the outcome measures were distributed around the midpoint, indicating robust results, as shown in Figure 3. Since there were 11 studies involved, a sufficient number, we performed Egger’s test and funnel plot for the mortality rate. Our results indicated a *P*-value of .772; as *P* > .05, it suggests no publication bias in the study, as shown in Figure 4.

#### 3.2.2. Duration of ECMO

**Figure 5.**
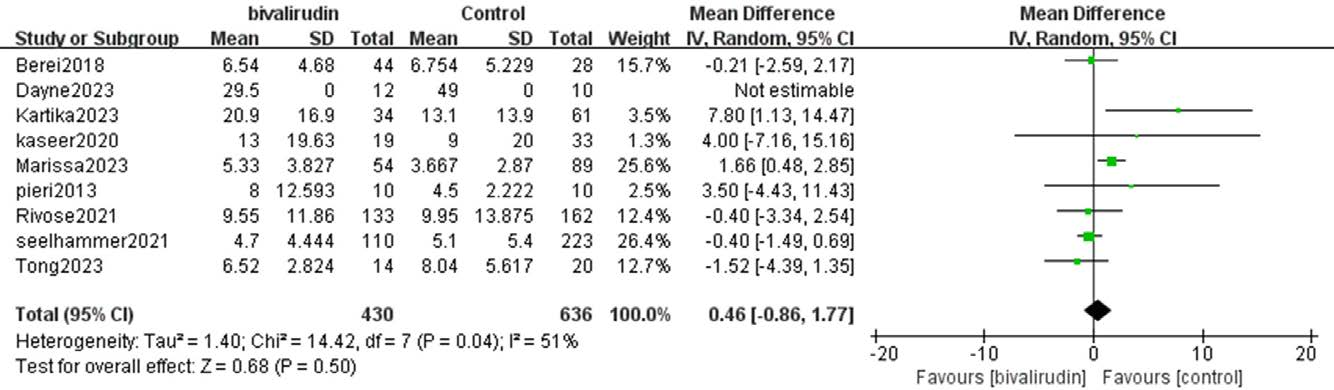
Forest plot comparing ECMO duration between bivalirudin and heparin groups. The plot displays mean differences (MD) and 95% confidence intervals (CIs) for ECMO duration across included studies. A random-effects model was used to account for study heterogeneity. CI = confidence interval, ECMO = extracorporeal membrane oxygenation, MD = mean differences, SD = square deviation.

There was moderate heterogeneity among the included studies (*I*² = 51%, *P* = .04), which may reflect variations in patient populations, treatment protocols, and institutional ECMO practices. While sensitivity analyses were conducted, further exploration using meta-regression could help identify sources of variability. The meta-analysis results showed a slightly longer ECMO duration in the bivalirudin group; however, this difference was not statistically significant (mean difference  = 0.46, 95% CI [−0.86, 1.77], *Z* = 0.68, *P* = .5), as shown in Figure 5.

#### 3.2.3. ECMO decannulation success rate

**Figure 6.**
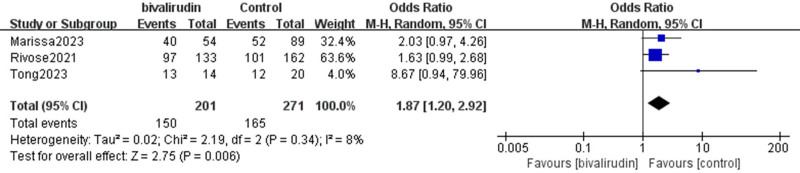
Forest plot comparing ECMO decannulation success rates between bivalirudin and heparin groups. The plot presents odds ratios (ORs) and 95% confidence intervals (CIs) for decannulation success across included studies. A random-effects model was applied to synthesize effect estimates. CI = confidence intervals, ECMO = extracorporeal membrane oxygenation, OR = odds ratios.

Our results indicate that 3 studies analyzed the impact of bivalirudin versus heparin anticoagulation on the ECMO decannulation success rate in ECMO patients. There was no significant heterogeneity among the studies (*I*² = 8%, *P* = .34). The meta-analysis results showed that the ECMO decannulation success rate was higher in the bivalirudin group compared to the control group, with a significance level of *P* < .05, indicating a statistically significant difference between the 2 groups (OR = 1.87, 95% CI [1.2, 2.92], *Z* = 2.75, *P* = .006), as shown in Figure 6.

#### 3.2.4. APTT

**Figure 7.**
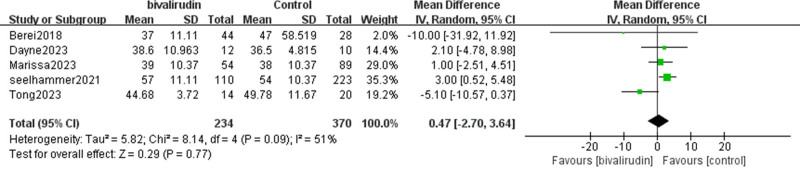
Forest plot comparing activated partial thromboplastin time (APTT) between bivalirudin and heparin groups. Mean differences (MD) with 95% confidence intervals (CIs) are displayed for APTT values across included studies. The random-effects model accounts for inter-study variability. APTT = activated partial thromboplastin time, CI = confidence interval, ECMO = extracorporeal membrane oxygenation, MD = mean differences, SD = square deviation.

According to the statistics, we found that 5 studies described the impact of bivalirudin versus heparin anticoagulation on APTT. There was relatively high heterogeneity among the studies (*I*² = 51%, *P* = .09). This variability may, in part, reflect differences in institutional anticoagulation monitoring protocols – such as target APTT ranges, testing intervals, or use of adjunctive monitoring strategies – which were not uniformly reported. The final meta-analysis results showed that the APTT level in the experimental group was slightly higher than that in the control group, but this difference was not statistically significant (mean difference  = 0.47, 95% CI [−2.7, 3.64], *Z* = 0.29, *P* = .77), as shown in Figure 7.

#### 3.2.5. Bleeding event rate

**Figure 8.**
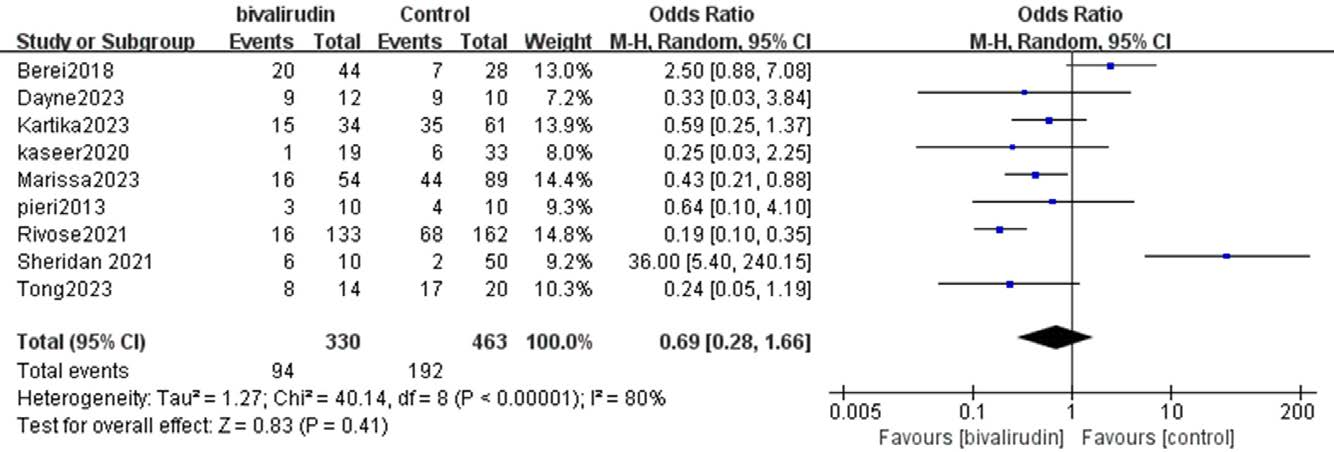
Forest plot comparing bleeding event rates between bivalirudin and heparin groups. Odds ratios (ORs) and 95% confidence intervals (CIs) are shown for each included study. A random-effects model was used to account for substantial heterogeneity among studies. CI = confidence interval, ORs = odds ratios.

Our study indicated that 9 studies analyzed the impact of bivalirudin versus heparin anticoagulation on the bleeding event rate in patients undergoing ECMO. The analysis revealed substantial heterogeneity among the studies (*I*² = 80%, *P* < .00001), indicating a wide variation in bleeding event rates across studies. This heterogeneity may be attributed to differences in anticoagulation regimens (e.g., dosage or monitoring strategy), patient comorbidities, and institutional protocols. Although sensitivity analysis was performed, future meta-regression or stratified analyses could further elucidate the sources of this heterogeneity. The pooled result suggested a trend toward fewer bleeding events in the bivalirudin group, but this was not statistically significant (OR = 0.69, 95% CI [0.28, 1.66], *Z* = 0.83, *P* = .41), as shown in Figure 8.

#### 3.2.6. Rate of thrombotic events

**Figure 9.**
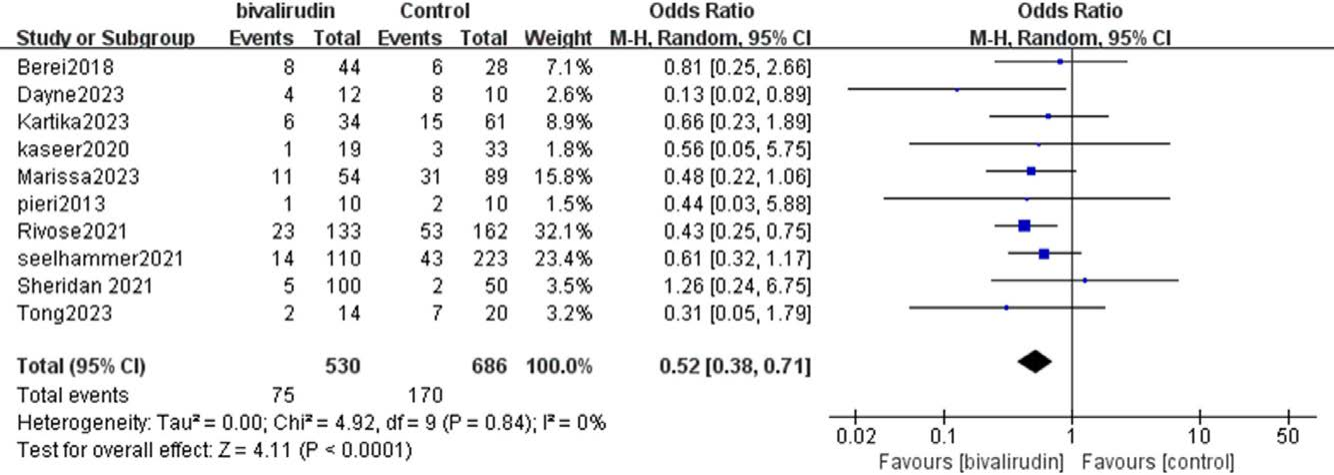
Forest plot comparing thrombotic event rates between bivalirudin and heparin groups. The plot displays odds ratios (ORs) and 95% confidence intervals (CIs) for the occurrence of thrombotic events. A random-effects model was applied, with low heterogeneity observed across included studies. CI = confidence interval, OR = odds ratios.

Our study revealed that 10 studies analyzed the impact of bivalirudin versus heparin anticoagulation on the rate of thrombotic events in patients undergoing ECMO. The final results showed no significant heterogeneity among the studies (*I*² = 0%, *P* = .84). Our findings demonstrated that compared to heparin anticoagulation, bivalirudin was associated with a lower rate of thrombotic events in ECMO patients, and this difference was statistically significant (OR = 0.52, 95% CI [0.38, 0.71], *Z* = 4.11, *P* < .0001), as shown in Figure 9.

#### 3.2.7. Platelet transfusion volume

**Figure 10.**
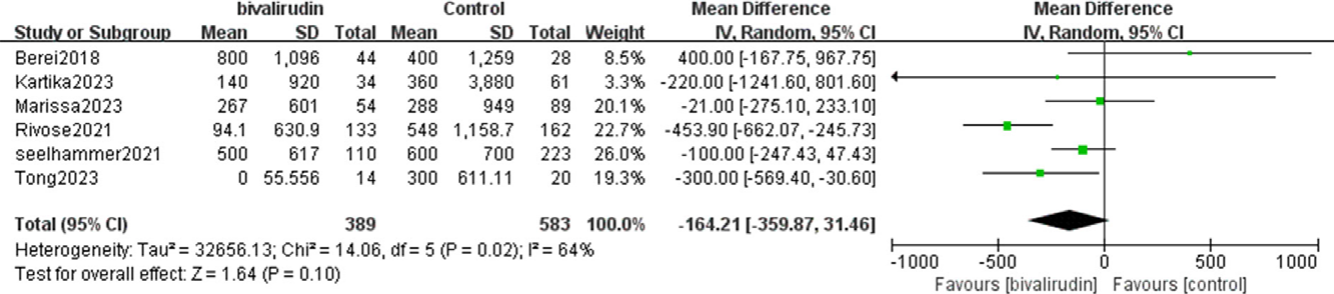
Forest plot comparing platelet transfusion volumes between bivalirudin and heparin groups. Mean differences (MD) with 95% confidence intervals (CIs) are shown for each included study. A random-effects model was used to account for heterogeneity in transfusion practices and reporting. CI = confidence interval, MD = mean differences, SD = square deviation.

Our study indicated that 6 studies analyzed the impact of bivalirudin versus heparin anticoagulation on platelet transfusion volume in patients undergoing ECMO. Our findings showed a nonsignificant trend toward lower platelet transfusion volume in the bivalirudin group compared to the heparin group. However, this result should be interpreted with caution due to moderate heterogeneity among the studies (*I*² = 64%, *P* = .02) and potential variability in transfusion thresholds across institutions. Differences in institutional protocols, clinician judgment, and patient-specific bleeding risk may influence transfusion practices, thereby limiting the comparability of this outcome (OR = −164.21, 95% CI [−359.84, 31.46], *Z* = 1.64, *P* = .1), as shown in Figure 10.

## 4. Discussion

ECMO is an advanced technology in intensive care units for rescuing critically ill patients and is commonly used to save the lives of patients with severe cardiopulmonary failure. It is categorized into different modes, such as VV-ECMO and VA-ECMO, based on the different methods of catheterization.^[20]^ The principle of ECMO involves using a machine pump to withdraw blood from the body, passing the blood through an oxygenator where it can be oxygenated and have carbon dioxide removed before being returned to the patient’s body.^[21]^ This allows patients with complete heart and lung damage to receive significant assistance. Due to the clear support ECMO provides for respiratory and circulatory function, it is widely used in intensive care units. As ECMO requires blood drainage, it inevitably leads to coagulation, necessitating anticoagulant therapy, with heparin being a commonly used anticoagulant; Lim J.Y. et al’s study indicated that in patients receiving VA-ECMO, heparin compared to sodium bivalirudin can significantly reduce the risk of bleeding.^[22]^ Although heparin is effective, it has some drawbacks. Firstly, heparin heavily relies on antithrombin III, leading to poor anticoagulant effects in certain patients. Secondly, heparin typically causes platelet depletion, which may stem from antibodies forming complexes between platelet factor 4 and polyanions, easily inducing immune coagulation factor disorders, thereby causing platelet reduction.^[23]^ Therefore, it is necessary to research alternative medications to heparin to improve the treatment effectiveness of ECMO patients and reduce the mortality rate.

Bivalirudin is an anticoagulant drug commonly used for preventing and treating thrombosis. It is a DTI that reduces clot formation by inhibiting thrombin activity in the blood.^[24]^ Bivalirudin’s anticoagulant advantages include predictable anticoagulant effects, lower risk of bleeding, and no need for regular monitoring of clotting indicators, among others. With these significant advantages, bivalirudin can be utilized for anticoagulant therapy in ECMO patients, as Erdoes G. et al’s study confirmed its superior anticoagulant effect in extracorporeal circulation surgery.^[25]^ Both bivalirudin and heparin have their own advantages, but there is divergence on which anticoagulant drug is more effective and safer for ECMO patients. Some believe that heparin is better due to its specific antagonist protamine sulfate^[26]^; others consider bivalirudin superior because it is a DTI with good predictability, simultaneously eliminating heparin-induced platelet decrease.^[27]^ Our study involved 1360 ECMO patients, dividing them into bivalirudin and heparin groups, with bivalirudin as the experimental group and heparin as the control group. We analyzed factors such as mortality rate, duration of ECMO, successful ECMO decannulation rate, thrombotic event rate, bleeding event rate, APTT value, and platelet transfusion volume between the 2 groups using software.

In both the ECMO duration and bleeding event analyses, we observed moderate to high statistical heterogeneity. This variation likely stems from differences in patient demographics, severity of illness, ECMO configurations, and variations in anticoagulation protocols (e.g., bolus vs continuous infusion, dosing strategies). While sensitivity analyses were performed to assess result robustness, future studies using meta-regression may help identify specific moderators contributing to this heterogeneity. A standardized approach to anticoagulation and more homogeneous reporting across studies would be valuable in reducing such variability.

Our subgroup analysis indicated a potential mortality benefit with bivalirudin in the VV-ECMO subgroup; however, the statistical significance was marginal and may not reflect a robust clinical effect. Moreover, the heterogeneity between VV and VA-ECMO in terms of patient profiles, underlying conditions, and disease severity further complicates direct comparison. As such, caution is warranted when interpreting subgroup results, and overgeneralization of bivalirudin’s benefit across all ECMO types should be avoided. In particular, underlying pathology – such as ARDS in VV-ECMO versus cardiogenic shock in VA-ECMO – may significantly influence treatment response. Future research should stratify outcomes by underlying disease states to enhance clinical applicability. The results from the subgroup analysis of VV-ECMO versus VA-ECMO patients may be influenced by several factors. VV-ECMO, which is mainly used for respiratory failure, and VA-ECMO, which provides both cardiac and respiratory support, represent 2 distinct patient populations with varying disease severity and treatment needs. This may explain the observed differences in mortality rates between the 2 groups, with no statistically significant difference found in either subgroup. The heterogeneity observed in these subgroup analyses could be due to variations in ECMO mode, patient characteristics, and disease severity. Further studies should explore the impact of anticoagulant choice within each ECMO subgroup to determine if different strategies are more appropriate for VV versus VA patients.

Our final results showed that there was no significant difference in the duration of ECMO between the bivalirudin group and the control group, indicating the safety of bivalirudin and no extension of ECMO treatment time.

Our meta-analysis final results show that the decannulation success rate of the bivalirudin group’s ECMO is higher than that of the control group’s ECMO. The reason for this is that bivalirudin has a smaller impact on platelets compared to heparin, which does not damage platelets and helps maintain normal clotting function, resulting in a higher decannulation success rate. This aligns with the results of some studies.

The final result of our meta-analysis showed that there is no significant difference in APTT values between the bivalirudin group and the control group, proving that bivalirudin’s anticoagulant effect is stable with minimal impact on the clotting system.

Our results confirm that there is no significant difference in the occurrence rate of bleeding events between the bivalirudin group and the heparin group, demonstrating that using bivalirudin for anticoagulation does not lead to an increase in bleeding events, making bivalirudin anticoagulation safe and reliable.

Our results also indicate that the occurrence rate of thrombotic events is lower in the bivalirudin group compared to the heparin group; this is similar to the results of Navaei A. et al’s study.^[28]^ The possible reason is that bivalirudin is a DTI that does not rely on antithrombin III, resulting in a more stable anticoagulant effect compared to heparin.

Although the pooled analysis revealed a nonsignificant reduction in platelet transfusion volume with bivalirudin, the clinical interpretation of this finding is limited by inter-study variability and moderate heterogeneity. Notably, transfusion decisions are influenced by a range of factors including institutional protocols, clinical judgment, platelet thresholds, and bleeding risk, all of which vary significantly across centers. As such, this outcome measure may lack standardization across studies. Further research with uniform transfusion criteria and standardized reporting is warranted to better evaluate the impact of anticoagulation on platelet utilization.

Previous studies, such as the meta-analysis by Wieruszewski P.M. et al,^[29]^ included a smaller number of studies and did not assess publication bias, which may have introduced bias in the findings. In contrast, our meta-analysis incorporates more recent, high-quality studies and provides a more comprehensive and up-to-date evaluation of bivalirudin’s effectiveness in ECMO patients.

The limitations of our study are multifaceted and must be acknowledged to ensure an accurate interpretation of the findings. First, inconsistencies in anticoagulation dosing protocols across studies – both in the heparin and bivalirudin groups—may introduce treatment bias. Second, significant heterogeneity was observed in outcomes such as ECMO duration (*I*² = 51%) and bleeding events (*I*² = 80%), which may stem from differences in patient populations, ECMO configurations, and institutional protocols. While sensitivity analyses were conducted, future studies should utilize meta-regression to explore sources of variability. Third, platelet transfusion thresholds varied across institutions, reflecting different clinical practices that likely influenced transfusion volume outcomes; this variability limits comparability across studies and should be standardized in future research. Fourth, anticoagulation monitoring protocols, including APTT targets and frequency, were inconsistently reported and may have impacted outcomes such as bleeding and thrombosis rates. Institutional-level differences, including staff experience, ECMO management protocols, and supportive care strategies – may independently affect both mortality and complication rates, highlighting the importance of transparent reporting in future studies. Lastly, our subgroup analyses comparing VV and VA-ECMO patients may oversimplify complex clinical scenarios. These modalities represent fundamentally distinct patient populations with unique pathophysiological profiles; therefore, outcomes should ideally be stratified by underlying pathology (e.g., ARDS vs cardiogenic shock) to enhance clinical relevance. A more rigorous, stratified approach is essential for future meta-analyses to better inform precision medicine in ECMO anticoagulation..

In our analysis, we observed a potential impact of the type of ECMO cannulation (VV vs VA) on mortality outcomes. Specifically, studies such as Dayne 2023 and Tong 2023^[9,11]^ focused exclusively on VV-ECMO patients, and while these studies contributed to the overall meta-analysis, the outcomes from these studies showed a reduced mortality rate that may not be generalizable to all ECMO patients, especially those undergoing VA-ECMO. Given that the weight of these VV-only studies in the overall analysis is relatively low, we believe it is important to clarify that the type of ECMO (VV vs VA) could significantly influence mortality outcomes, and this should be taken into consideration when interpreting the effect of anticoagulation choices.

Although our findings indicate a lower mortality rate with bivalirudin, particularly in VV-ECMO patients, it is crucial to note that mortality is influenced by multiple factors beyond anticoagulation therapy, including patient-specific variables, the underlying disease process, and the type of ECMO used. This nuanced understanding of the data helps highlight that while bivalirudin may offer benefits in terms of thrombotic events and decannulation success, the type of ECMO and other clinical factors may play a more substantial role in determining patient outcomes.

We recommend that future studies further explore the influence of ECMO mode (VV vs VA) on mortality and anticoagulation outcomes to better isolate the effects of anticoagulant therapy in the context of different ECMO modalities.

## 5. Conclusion

This study suggests that bivalirudin anticoagulation may offer certain advantages over traditional heparin in ECMO patients, including a lower incidence of thrombotic events and improved decannulation success rates. While subgroup analysis indicated a possible mortality benefit in VV-ECMO patients, this finding did not reach clear statistical significance and should be interpreted cautiously due to underlying clinical heterogeneity. Significant variability in ECMO duration and bleeding outcomes further underscores the influence of confounding factors, such as institutional differences in anticoagulation monitoring, transfusion thresholds, and patient selection. Future studies should adopt standardized protocols and stratify results based on ECMO modality and underlying pathology to ensure clinical applicability. These findings suggest that bivalirudin may be a more effective anticoagulant option in specific ECMO contexts, but stronger evidence from randomized trials is needed to validate these conclusions.

## Acknowledgments

Thank you for the support from Shenzhen Longgang District Second People’s Hospital in the research.

## Author contributions

**Conceptualization:** Wendian Yang.

**Data curation:** Wendian Yang, Yunnan Hu.

**Formal analysis:** Yunnan Hu.

**Funding acquisition:** Wendian Yang.

**Investigation:** Linchang Yu.

**Methodology:** Yunnan Hu, Linchang Yu.

**Project administration:** Wendian Yang, Yunnan Hu.

**Resources:** Yunnan Hu.

**Software:** Yunnan Hu.

**Supervision:** Wendian Yang, Yunnan Hu.

**Validation:** Wendian Yang.

**Writing** – **original draft:** Wendian Yang, Yunnan Hu.

**Writing** – **review & editing:** Wendian Yang, Yunnan Hu, Linchang Yu.

## Supplementary Material



## References

[1] Bartlett RH. The story of ECMO. Anesthesiology. 2024;140:578–84. 38349754 10.1097/ALN.0000000000004843

[2] Abrams D MacLaren G Lorusso R. Extracorporeal cardiopulmonary resuscitation in adults: evidence and implications. Intensive Care Med. 2022;48:1–15. 34505911 10.1007/s00134-021-06514-y PMC8429884

[3] Figueroa Villalba CA McMullan DM Reed RC Chandler WL. Thrombosis in extracorporeal membrane oxygenation (ECMO) circuits. ASAIO J. 2022;68:1083–92. 34860711 10.1097/MAT.0000000000001605

[4] Mazzeffi MA Tanaka K Roberts A. Bleeding, thrombosis, and transfusion with two heparin anticoagulation protocols in venoarterial ECMO patients. J Cardiothorac Vasc Anesth. 2019;33:1216–20. 30181084 10.1053/j.jvca.2018.07.045

[5] Hogan M Berger JS. Heparin-induced thrombocytopenia (HIT): review of incidence, diagnosis, and management. Vasc Med. 2020;25:160–73. 32195628 10.1177/1358863X19898253

[6] Selvadurai MV Favaloro EJ Chen VM. Mechanisms of thrombosis in heparin-induced thrombocytopenia and vaccine-induced immune thrombotic thrombocytopenia. Semin Thromb Hemost. 2023;49:444–52. 36706782 10.1055/s-0043-1761269

[7] Hsu E Moosavi L. Biochemistry, antithrombin III. In: StatPearls. StatPearls Publishing; 2023. 31424879

[8] Hamzah M Jarden AM Ezetendu C Stewart R. Evaluation of bivalirudin as an alternative to heparin for systemic anticoagulation in pediatric extracorporeal membrane oxygenation. Pediatr Crit Care Med. 2020;21:827–34. 32404633 10.1097/PCC.0000000000002384

[9] Diaz D Martinez J Bushman G Wolowich WR. Anticoagulation strategies in COVID-19 infected patients receiving ECMO support. J Extra Corpor Technol. 2023;55:121–9. 37682210 10.1051/ject/2023027 PMC10487306

[10] Uricchio MN Ramanan R Esper SA. Bivalirudin versus unfractionated heparin in patients with cardiogenic shock requiring venoarterial extracorporeal membrane oxygenation. ASAIO J. 2023;69:107–13. 35412480 10.1097/MAT.0000000000001723

[11] Tong Y Rouzhahong J Zhou W. Comparison of bivalirudin versus heparin in adult extracorporeal membrane oxygenation anticoagulant therapy: a retrospective case-control study. Int J Artif Organs. 2023;46:162–70. 36600413 10.1177/03913988221148763

[12] Kartika T Mathews R Migneco G. Comparison of bleeding and thrombotic outcomes in veno-venous extracorporeal membrane oxygenation: heparin versus bivalirudin. Eur J Haematol. 2024;112:566–76. 38088062 10.1111/ejh.14146 PMC11034845

[13] Berei TJ Lillyblad MP Wilson KJ Garberich RF Hryniewicz KM. Evaluation of systemic heparin versus bivalirudin in adult patients supported by extracorporeal membrane oxygenation. ASAIO J. 2018;64:623–9. 29076942 10.1097/MAT.0000000000000691

[14] Pieri M Agracheva N Bonaveglio E. Bivalirudin versus heparin as an anticoagulant during extracorporeal membrane oxygenation: a case-control study. J Cardiothorac Vasc Anesth. 2013;27:30–4. 23036625 10.1053/j.jvca.2012.07.019

[15] Seelhammer TG Bohman JK Schulte PJ Hanson AC Aganga DO. Comparison of bivalirudin versus heparin for maintenance systemic anticoagulation during adult and pediatric extracorporeal membrane oxygenation. Crit Care Med. 2021;49:1481–92. 33870916 10.1097/CCM.0000000000005033

[16] Rivosecchi RM Arakelians AR Ryan J. Comparison of anticoagulation strategies in patients requiring venovenous extracorporeal membrane oxygenation: heparin versus bivalirudin. Crit Care Med. 2021;49:1129–36. 33711003 10.1097/CCM.0000000000004944

[17] Sheridan EA Sekela ME Pandya KA Schadler A Ather A. Comparison of bivalirudin versus unfractionated heparin for anticoagulation in adult patients on extracorporeal membrane oxygenation. ASAIO J. 2022;68:920–4. 34669620 10.1097/MAT.0000000000001598

[18] Giuliano K Bigelow BF Etchill EW. Extracorporeal membrane oxygenation complications in heparin- and bivalirudin-treated patients. Critical Care Explorations. 2021;3:e0485. 34278315 10.1097/CCE.0000000000000485 PMC8280085

[19] Kaseer H Soto-Arenall M Sanghavi D. Heparin vs bivalirudin anticoagulation for extracorporeal membrane oxygenation. J Card Surg. 2020;35:779–86. 32048330 10.1111/jocs.14458

[20] Lim H. The physiology of extracorporeal membrane oxygenation: the Fick principle. Perfusion. 2023;38:236–44. 34961381 10.1177/02676591211055971

[21] Riley JB Searles BE Darling EM Oles DM Aiash H. The effectiveness of three different curricular models to teach fundamental ECMO specialist skills to entry level perfusionists. J Extra Corpor Technol. 2021;53:245–50. 34992314 10.1182/ject-2100008 PMC8717719

[22] Lim JY Kim JB Choo SJ Chung CH Lee JW Jung SH. Anticoagulation during extracorporeal membrane oxygenation; nafamostat mesilate versus heparin. Ann Thorac Surg. 2016;102:534–9. 27083248 10.1016/j.athoracsur.2016.01.044

[23] Arepally GM Cines DB. Pathogenesis of heparin-induced thrombocytopenia. Transl Res. 2020;225:131–40. 32417430 10.1016/j.trsl.2020.04.014 PMC7487042

[24] Valgimigli M Frigoli E Leonardi S. Radial versus femoral access and bivalirudin versus unfractionated heparin in invasively managed patients with acute coronary syndrome (MATRIX): final 1-year results of a multicentre, randomised controlled trial. Lancet. 2018;392:835–48. 30153988 10.1016/S0140-6736(18)31714-8

[25] Erdoes G Ortmann E Martinez Lopez De Arroyabe B Reid C Koster A. Role of bivalirudin for anticoagulation in adult perioperative cardiothoracic practice. J Cardiothorac Vasc Anesth. 2020;34:2207–14. 31521492 10.1053/j.jvca.2019.08.022

[26] Derbalah A Duffull S Newall F Moynihan K Al-Sallami H. Revisiting the pharmacology of unfractionated heparin. Clin Pharmacokinet. 2019;58:1015–28. 30850987 10.1007/s40262-019-00751-7

[27] Burša F Sklienka P Frelich M Jor O Ekrtová T Máca J. Anticoagulation management during extracorporeal membrane oxygenation-a mini-review. Medicina. 2022;58:1783. 36556985 10.3390/medicina58121783 PMC9782867

[28] Navaei A Kostousov V Teruya J. Is it time to switch to bivalirudin for ECMO anticoagulation? Front Med (Lausanne). 2023;10:1237601. 37671395 10.3389/fmed.2023.1237601 PMC10476497

[29] Wieruszewski PM Macielak SA Nei SD. Heparin versus bivalirudin for anticoagulation in adult extracorporeal membrane oxygenation: a systematic review and meta-analysis. ASAIO J. 2023;69:137–44. 36355803 10.1097/MAT.0000000000001808

