# Supplementary material for: Effectiveness and safety of bivalirudin anticoagulation therapy in adult patients receiving extracorporeal membrane oxygenation: A systematic review and meta-analysis
Source: Medicine (Baltimore). 2025 Aug 15;104(33):e42696. doi: 10.1097/MD.0000000000042696 (PMC12366896; doi:10.1097/MD.0000000000042696)
Supplement: Supplementary file 1 [file medi-104-e42696-s001.docx]

**Search approach.**

(("bivalirudin" [Supplementary Concept]) OR ((((((((((((((bivalirudin[Title/Abstract]) OR (Phe-Pro-Arg-Pro-(Gly[Title/Abstract])4-desulfohirudin-(53-64))) OR (Phe-Pro-Arg-Pro-(Gly)4-Asn-Gly-Asp-Phe-Glu-Glu-Ile-Pro-Glu-Glu-Tyr-Leu[Title/Abstract])) OR (Phe-Pro-Arg-Pro-(Gly)4-Asn-Gly-Asp-Phe-Glu-Glu-Ile-Pro-Glu-Glu-Tyr-Leu[Title/Abstract])) OR (Phe-Pro-Arg-Pro-(Gly)4 desulfato-Tyr63'-hirugen[Title/Abstract])) OR (Angiomax[Title/Abstract])) OR (Angiomax RTU[Title/Abstract])) OR (CTB-001[Title/Abstract])) OR (bivalirudin trifluoroacetate[Title/Abstract])) OR (BG 8967[Title/Abstract])) OR (BG8967[Title/Abstract])) OR (BG-8967[Title/Abstract])) OR (Hirulog[Title/Abstract])) OR (Hirulog-1[Title/Abstract]))) AND (("Extracorporeal Membrane Oxygenation"[Mesh]) OR ((((((((((((((((((extracorporeal membrane oxygenation[Title/Abstract]) OR (ECMO[Title/Abstract])) OR (extracorporeal life support[Title/Abstract])) OR (ECLS[Title/Abstract])) OR (Extracorporeal Membrane Oxygenations[Title/Abstract])) OR (ECMO Treatment[Title/Abstract])) OR (ECMO Treatments[Title/Abstract])) OR (ECLS Treatment[Title/Abstract])) OR (ECLS Treatments[Title/Abstract])) OR (ECMO Extracorporeal Membrane Oxygenation[Title/Abstract])) OR (Extracorporeal Life Support[Title/Abstract])) OR (Extracorporeal Life Supports[Title/Abstract])) OR (Venoarterial ECMO[Title/Abstract])) OR (Venoarterial ECMOs[Title/Abstract])) OR (Venoarterial Extracorporeal Membrane Oxygenation[Title/Abstract])) OR (Venovenous ECMO[Title/Abstract])) OR (Venovenous ECMOs[Title/Abstract])) OR (Venovenous Extracorporeal Membrane Oxygenation[Title/Abstract]))).
